# Sustainable Synthesis of Calcium Propionate from Cockle Shell Biowaste for Food Additive Production

**DOI:** 10.3390/ijms27114955

**Published:** 2026-05-29

**Authors:** Chaowared Seangarun, Banjong Boonchom, Somkiat Seesanong, Wimonmat Boonmee, Sirichet Punthipayanon, Nongnuch Laohavisuti, Pesak Rungrojchaipon

**Affiliations:** 1 Material Science for Environmental Sustainability Research Unit, School of Science, King Mongkut’s Institute of Technology Ladkrabang, Bangkok 10520, Thailand; chaowared@gmail.com (C.S.); wimonmat.bo@kmitl.ac.th (W.B.); sirichet@g.swu.ac.th (S.P.); pesak.ru@kmitl.ac.th (P.R.); 2 Department of Chemistry, School of Science, King Mongkut’s Institute of Technology Ladkrabang, Bangkok 10520, Thailand; 3 Municipal Waste and Wastewater Management Learning Center, School of Science, King Mongkut’s Institute of Technology Ladkrabang, Bangkok 10520, Thailand; 4 Office of Administrative Interdisciplinary Program on Agricultural Technology, School of Agricultural Technology, King Mongkut’s Institute of Technology Ladkrabang, Bangkok 10520, Thailand; somkiat.se@kmitl.ac.th; 5 Department of Biology, School of Science, King Mongkut’s Institute of Technology Ladkrabang, Bangkok 10520, Thailand; 6 Department of Sports Science, Faculty of Physical Education, Srinakharinwirot University, Bangkok 10110, Thailand; 7 Thailand Association of Mixed Martial Arts, 431/19, Rajchadapisek Road, Bangkok 10120, Thailand

**Keywords:** calcium propionate, cockle shell, calcium carbonate, biowaste refinery, food preservative

## Abstract

Calcium propionate (Ca(CH_3_CH_2_COO)_2_) was successfully synthesized from cockle shell biowaste through a reaction with propionic acid at concentrations of 80%, 90%, and 99%, valorizing seafood processing biowaste as a renewable calcium source in support of circular economy principles. The synthesis was conducted at ambient temperature with a fixed CaCO_3_: propionic acid molar ratio of 1:2, enabling rapid reaction completion without external heating or complex purification steps. The prepared samples were characterized by FTIR, XRD, TGA, and SEM techniques, which confirmed the formation of calcium propionate monohydrate (Ca(CH_3_CH_2_COO)_2_·H_2_O), while XRF confirmed more than 97 wt% CaO across all samples with non-toxic impurities corresponding to compositional requirements for food additive calcium propionate (E282). The sample prepared using 80% propionic acid exhibited the highest yield (90.24%) and soluble percentage (98.23%). The proposed approach demonstrates an effective valorization of cockle shell waste into a food additive, calcium propionate, offering advantages in terms of sustainability, cost efficiency, and scalability, and highlighting its strong potential for industrial food additive production within a circular economy framework.

## 1. Introduction

The increasing demand for calcium-based compounds in food, pharmaceutical, and industrial applications has stimulated continuous efforts to develop sustainable and cost-effective production routes. Among these compounds, calcium propionate (Ca(C_2_H_5_COO)_2_) has attracted considerable attention for its widespread use as a food preservative [1,2]. Calcium propionate is widely employed as an antimicrobial agent to inhibit the growth of molds and bacteria, thereby extending shelf life and maintaining food quality without adversely affecting sensory properties [3]. Owing to its recognized safety, it has been approved for food applications by regulatory authorities such as the U.S. Food and Drug Administration (FDA) and the European Food Safety Authority (EFSA), and is commonly used in bread, cheese, and animal feed products [4,5]. With increasing global demand, the market volume of calcium propionate was reported at approximately 463,250 metric tons in 2022 and is projected to reach about 603,100 metric tons by 2030, reflecting its growing industrial and commercial significance [6].

Conventionally, the industrial production of calcium propionate is generally based on the neutralization of propionic acid with calcium hydroxide or calcium carbonate derived from mined limestone [7]. Many reported methods for transforming limestone into calcium salts require high-temperature calcination, prolonged reaction times, or the use of strong inorganic acids, which may compromise environmental sustainability and economic feasibility [8]. In the previous study by Mahmood et al. [9], limestone was treated with hydrochloric acid (HCl) and nitric acid (HNO_3_) to remove impurities before conversion to calcium propionate. However, this approach increases production costs and generates acidic wastewater, which may pose environmental concerns. The biological production of calcium propionate has been proposed as a more sustainable alternative. In such processes, calcium propionate is produced via anaerobic fermentation using propionate-producing microorganisms, with substrates such as glucose, glycerol, lactic acid, or acetic acid [10]. The biological route generally yields less than conventional chemical synthesis and still relies on calcium sources from mining during processing, which limits its overall sustainability [6]. Due to these limitations, eggshell-derived calcium carbonate has been widely investigated as a low-cost calcium source for producing calcium propionate via both chemical and biotechnological routes [11,12]. However, studies on the utilization of marine shell waste, such as cockle shells, for calcium propionate production remain scarce.

In parallel, the seafood-processing industry generates large quantities of shell waste, particularly mollusk shells, which are also mainly composed of calcium carbonate (CaCO_3_) [13]. Improper disposal of these wastes causes environmental issues such as odor emission, landfilling problems, and coastal pollution [14]. Among various types of mollusk shell waste, cockle shells constitute a major fraction generated in large quantities, contributing significantly to environmental burdens when discarded in landfills, with annual production from fishery waste exceeding 0.4 million tons [15]. Therefore, increasing attention has been directed toward the recycling and valorization of cockle shell waste as effective approaches for resource recovery and environmental sustainability [16]. In addition, biogenic calcium sources from cockle shells offer high calcium content (98–99%) and low cost, demonstrating their potential as an attractive alternative calcium source for producing calcium-based food additives [17].

According to our previous studies, the direct reaction of highly concentrated acids with shell waste has enabled the synthesis of calcium phosphate [18], calcium acetate [19], calcium lactate [20], and calcium citrate [21] through low-cost, rapid, and environmentally friendly processes. In all cases, CaCO_3_ reacts with the corresponding acid to produce the calcium salt, CO_2_, and H_2_O through a similar neutralization pathway. However, the resulting calcium compounds may exhibit different crystallization behaviors and hydration states depending on their molecular structures and physicochemical characteristics. This reaction provides a simple and potentially green approach to producing calcium propionate from a biological calcium source for food-additive applications. Calcium food additive compounds have different characteristic properties depending on the raw material used, the preparation method, and the controlled conditions (time, pH, pressure, temperature, mixing rate, etc.) [18,19,20,21]. Therefore, the objective of this study is to develop a rapid, low-cost, and environmentally friendly synthesis of calcium propionate from cockle-shell biowaste as a renewable calcium source, using propionic acid as the reactant. The proposed method aims to minimize processing steps, eliminate harsh chemicals, and reduce energy consumption, thereby offering a sustainable route for producing calcium propionate for food additive applications [11]. The physicochemical characteristics of the synthesized product are evaluated and compared with those of calcium propionate prepared in previous studies to assess its potential applicability in the food industry. This work demonstrates a green, sustainable valorization pathway to transform this marine biowaste into value-added products, thereby enhancing resource efficiency, reducing waste generation, and supporting the transition toward a circular economy.

## 2. Results and Discussion

### 2.1. Thermal Decomposition of CPN

**Figure 1:**
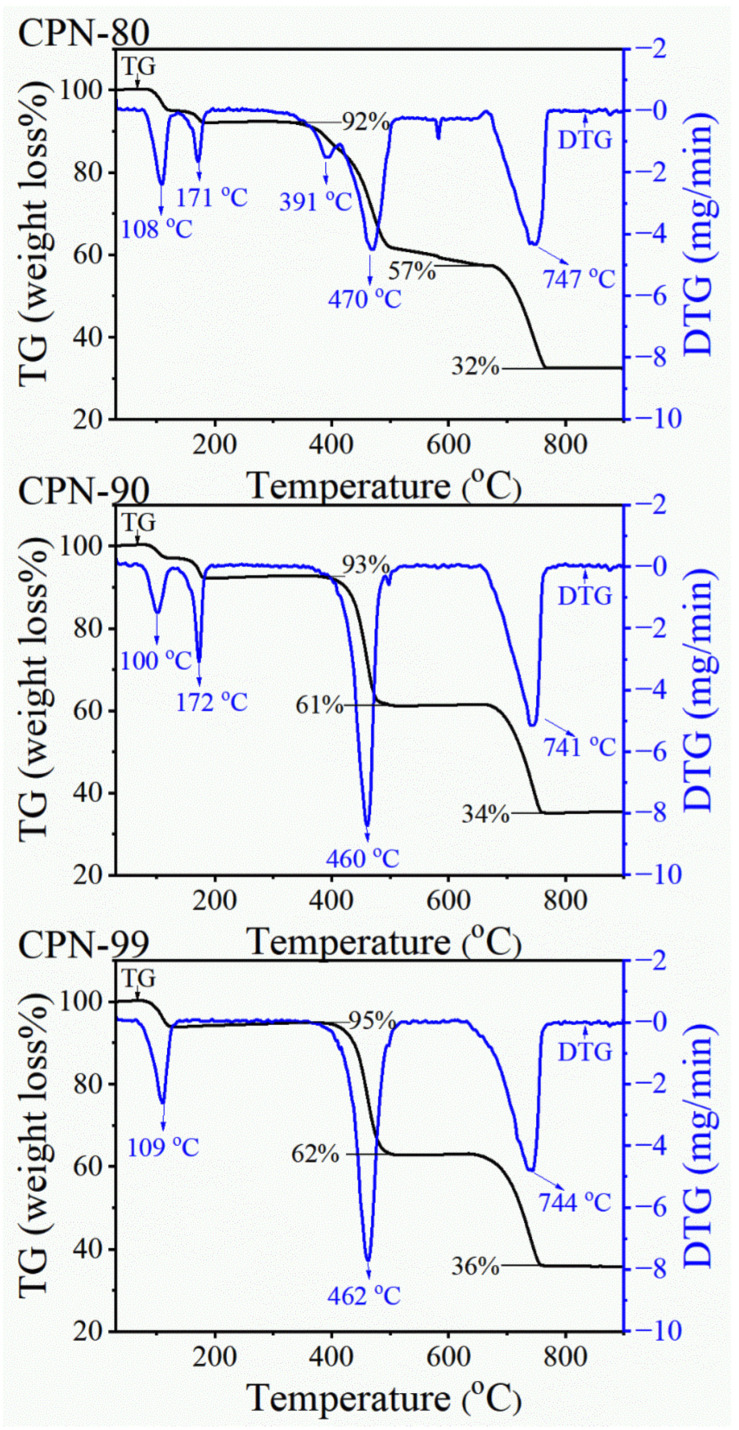
TG and DTG curves of calcium propionate derived from cockle shells using propionic acid with different concentrations (80%, 90%, and 99%), denoted as CPN-80, CPN-90, and CPN-99, respectively.

The thermal decomposition behavior of calcium propionate samples prepared using propionic acid concentrations of 80%, 90%, and 99%, denoted as CPN-80, CPN-90, and CPN-99, respectively, was evaluated by thermogravimetric analysis (TGA) under a nitrogen atmosphere from room temperature to 800 °C, and the results are presented in Figure 1. All three samples exhibited similar multi-stage decomposition profiles, consistent with the thermal behavior reported for calcium propionate in the previous work [22]. The first weight loss step occurs below approximately 200 °C, with a mass loss of about 5–8%wt, which is attributed to the removal of physically adsorbed moisture and hydrated water associated with calcium propionate monohydrate (Ca(CH_3_CH_2_COO)_2_·H_2_O). This step is accompanied by DTG peaks between 30 and 200 °C, consistent with dehydration behavior reported for hydrated calcium propionate analyzed by Zaidi et al. [22], resulting in the anhydrous form of calcium propionate, which corresponds to Equation (1):

Ca(CH_3_CH_2_COO)_2_·H_2_O → Ca(CH_3_CH_2_COO)_2_ + H_2_O (1)

The second decomposition step occurs in the temperature range of approximately 300–500 °C, corresponding to a weight loss of about 32–35%wt. This stage is associated with the thermal decomposition of propionate groups, resulting in the release of 3-pentanone (C_2_H_5_COC_2_H_5_) and the formation of calcium carbonate as an intermediate solid product, corresponding to Equation (2) [22].

Ca(CH_3_CH_2_COO)_2_ → CaCO_3_ + C_2_H_5_COC_2_H_5_ (2)

The third decomposition step occurs between approximately 650 and 800 °C, with an additional mass loss of around 25–27%wt. This stage is attributed to the decomposition of calcium carbonate with the evolution of CO_2_, corresponding to the thermal decomposition of calcium propionate in the previous work according to Equation (3) [23].

CaCO_3_ → CaO + CO_2_ (3)

The residual mass remaining at 800 °C is approximately 32–36%wt for all samples, which is consistent with the formation of CaO as the final inorganic residue. Although the overall TGA and DTG profiles are comparable for all samples, the CPN-80 exhibits a slightly higher onset temperature for the main decomposition step of propionate groups (470 °C), indicating higher thermal stability than the CPN90 and CPN-99 (460 and 462 °C, respectively), and higher than the thermal decomposition of commercial grade calcium propionate (466 °C) reported by Zaidi et al. [22].

### 2.2. Functional Groups of CPN

**Figure 2:**
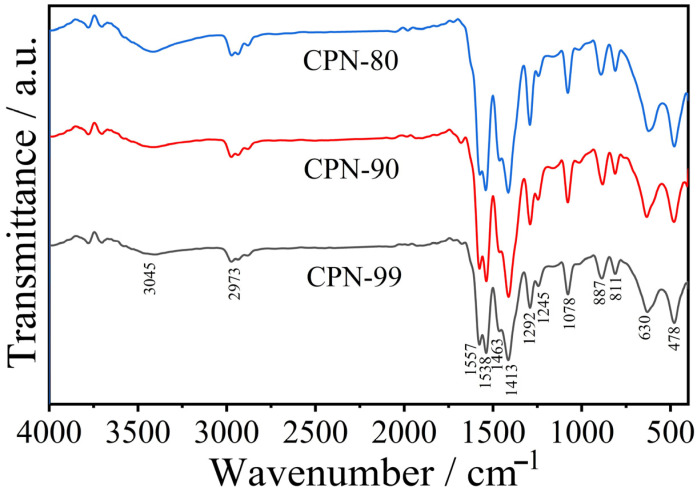
Fourier transform infrared (FTIR) spectra of calcium propionate derived from cockle shells using propionic acid with different concentrations (80%, 90%, and 99%), denoted as CPN-80, CPN-90, and CPN-99, respectively.

The Fourier transform infrared (FTIR) spectra of calcium propionate samples prepared using propionic acid concentrations of 80%, 90%, and 99%, denoted as CPN-80, CPN-90, and CPN-99, respectively, are shown in Figure 2. The FTIR spectra of the CPN-80, CPN-90, and CPN-99 samples exhibit similar absorption patterns, indicating that all samples contain the same functional groups. In all samples, the characteristic absorption bands of carboxylate groups (–COO^−^) were clearly observed. The asymmetric stretching vibration of the carboxylate group appeared in the region of approximately 1550–1600 cm^−1^, while the corresponding symmetric stretching vibration was detected around 1400–1450 cm^−1^. The presence of these two strong bands, together with the absence of the C=O stretching band of propionic acid (1700–1720 cm^−1^), confirms the formation of calcium propionate. Additionally, the absorption bands observed in the range of 2850–3000 cm^−1^ are attributed to the C–H stretching vibrations of CH_3_ and CH_2_ groups. A broad, weak band centered around 3400 cm^−1^ can be assigned to hydrated water, which is commonly observed in calcium salts prepared from shell wastes and has also been reported in our previous studies on calcium acetate and calcium lactate [19,20]. Importantly, no additional absorption bands corresponding to other functional groups were detected in any spectra. The FTIR results confirm that the CPN-80, CPN-90, and CPN-99 samples possess the same functional groups of calcium propionate monohydrate (Ca(CH_3_CH_2_COO)_2_·H_2_O), consistent with the FTIR spectra of commercial calcium propionate reported in the previous research [22].

### 2.3. Crystal Structure of CPN

**Figure 3:**
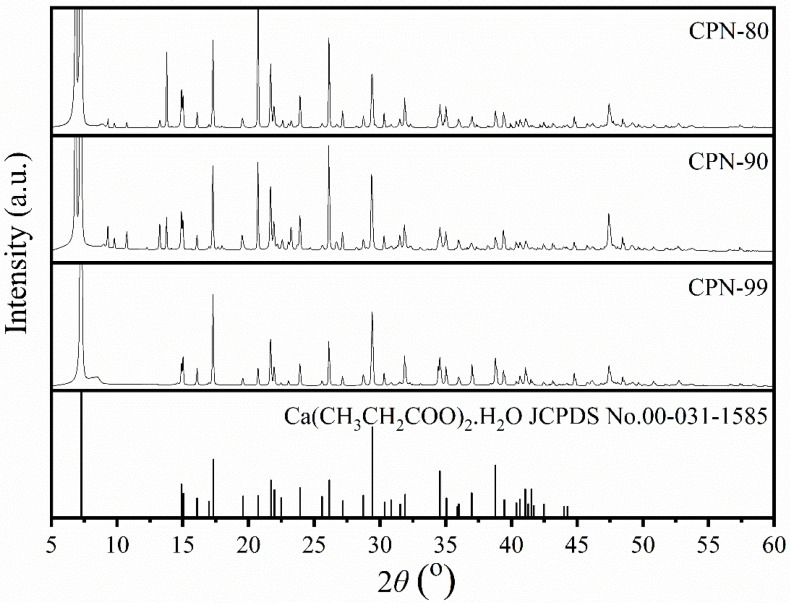
X-ray diffraction (XRD) patterns of calcium propionate derived from cockle shells using propionic acid with different concentrations (80%, 90%, and 99%), denoted as CPN-80, CPN-90, and CPN-99, respectively.

The X-ray diffraction (XRD) patterns of calcium propionate samples synthesized using propionic acid concentrations of 80%, 90%, and 99%, denoted as CPN-80, CPN-90, and CPN-99, respectively, are compared with the standard diffraction data of calcium propionate monohydrate (Ca(CH_3_CH_2_COO)_2_·H_2_O, JCPDS No. 00-031-1585), as shown in Figure 3. For all samples, the major diffraction peaks are in good agreement with the characteristic reflections of calcium propionate monohydrate (Ca(CH_3_CH_2_COO)_2_·H_2_O), indicating that this compound is the predominant crystalline phase in the synthesized products [24]. In addition, the CPN-80 and CPN-90 samples exhibit several weak additional diffraction peaks that are present in the standard JCPDS No. 00-031-1584, corresponding to the anhydrous form of calcium propionate (Ca(CH_3_CH_2_COO)_2_) [24]. These results confirm the formation of calcium propionate compounds in all synthesized samples.

### 2.4. Morphologies of CPN

**Figure 4:**
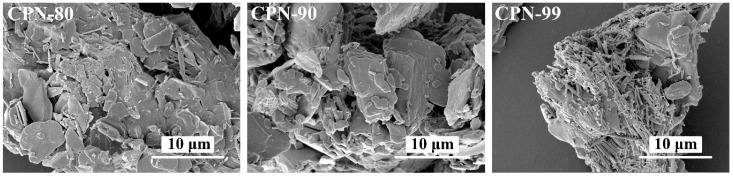
SEM images of calcium propionate derived from cockle shells using propionic acid with different concentrations (80%, 90%, and 99%), denoted as CPN-80, CPN-90, and CPN-99, respectively.

The SEM images of calcium propionate samples prepared using propionic acid concentrations of 80%, 90%, and 99%, denoted as CPN-80, CPN-90, and CPN-99, respectively, are shown in Figure 4. The SEM images were recorded at 5000× magnification after gold coating. The morphologies of the CPN-80, CPN-90, and CPN-99 samples show plate-like microparticles with different particle sizes ranging from around 2–10 μm. In addition, the morphology of the CPN-99 sample exhibits small timber-like particles on its surface. This observation is consistent with the previous report, which prepared calcium propionate from limestone and exhibited a similar plate-like morphology and particle size [9]. Notably, timber-like microparticles observed on the surface can be attributed to residual aragonite from the cockle shell-derived CaCO_3_, which is similar to the morphologies of calcium compounds from cockle shells reported by Seesanong et al. [25].

### 2.5. Purities of CPN

The chemical compositions of the synthesized calcium propionate samples prepared with propionic acid concentrations of 80%, 90%, and 99%, denoted CPN-80, CPN-90, and CPN-99, respectively, were determined by X-ray fluorescence (XRF) analysis, as shown in Table 1. The results indicate that all samples are predominantly composed of calcium, expressed as CaO, with contents ranging from 97.2 to 97.5 wt%. Minor amounts of oxide impurities, including Na_2_O, MgO, Al_2_O_3_, SiO_2_, P_2_O_5_, SO_3_, Fe_2_O_3_, and SrO, were detected at lower than 3%wt in all samples. The XRF results demonstrate that all synthesized calcium propionate samples possess high calcium purity with non-toxic impurities, in line with the general compositional requirements for food-grade calcium propionate (E282) [5]. However, for use as a food additive, calcium propionate needs to be further purified to over 99%. The calcium propionate compounds obtained in this study will be further purified.

### 2.6. Preparation Optimization Results

The preparation optimization results of calcium propionate synthesized using propionic acid with different concentrations (80%, 90%, and 99%), denoted as CPN-80, CPN-90, and CPN-99, respectively, are summarized in terms of reaction temperature, reaction time, product yield, and soluble percentage, as presented in Table 2. All experiments were conducted using a fixed CaCO_3_:propionic acid molar ratio of 1:2, as described in the Materials and Methods section. The reaction was considered complete when CO_2_ bubble formation completely ceased, indicating full consumption of CaCO_3_. CPP-80 exhibited the longest reaction time, whereas the CPP-99 sample reached completion within the shortest duration. This behavior can be attributed to higher acid concentration and enhanced exothermic reaction. The reaction proceeded efficiently at ambient temperature for all samples, with only a slight increase in temperature due to the exothermic reaction. No external heating was required, highlighting the process’s low-energy, environmentally benign nature. In terms of product yield, the CPN-80 sample exhibited the highest percentage yield at 90.24%, followed by 83.09% and 78.55% for the CPN-90 and CPN-99 samples, respectively. This result reflects the effective formation of calcium propionate monohydrate, accompanied by an increase in molecular weight from 100 g/mol for CaCO_3_ to 186.22 g/mol for calcium propionate monohydrate (Ca(CH_3_CH_2_COO)_2_·H_2_O), confirming the successful progression of the reaction. The soluble percentages of the calcium propionate samples, CPN80, CPN90, and CPN99, were 98.23%, 96.54%, and 91.81%, respectively. The obtained solubility values are consistent with the corresponding yields, indicating a high degree of conversion from CaCO_3_ to calcium propionate due to the high solubility of calcium propionate, which is classified as a freely water-soluble calcium salt, facilitating complete dissolution in aqueous systems, in contrast to calcium carbonate, which is classified as a low solubility compound [26,27].

Overall, the optimization results demonstrate that increasing propionic acid concentration enhances reaction efficiency by reducing reaction time and improving product yield. These findings are consistent with the synthesis of shell-derived calcium acetate [19], calcium lactate [20], and calcium citrate [21] in our previous reports, where acid concentration played a key role in determining synthesis performance. The results of the CPN-80 sample confirm that 80% propionic acid is the optimal condition in this research for the low-cost, environmentally friendly production of calcium propionate from cockle shell biowaste, yielding the highest yield and solubility. Notably, the maximum yield obtained in this study (90.24%) at an acid concentration of 80% is higher than those reported in previous studies using limestone as a calcium source, such as Mahmood et al. [9], which achieved a maximum yield at 85% using 15% acid concentration, and Rajendran et al. [6], which reported a maximum yield at 86% using 10% acid concentration. In addition, the use of highly concentrated propionic acid in the present study significantly shortened the reaction time compared with previous reports [6], while simultaneously improving the overall product yield. The enhanced propionic acid concentration also enabled the synthesis to proceed effectively without additional external heating, thereby simplifying the production process. Compared with conventional limestone processing, shell-derived CaCO_3_ may require fewer purification steps while simultaneously helping reduce environmental problems associated with shell waste disposal. Previous life cycle assessment studies have reported that the major environmental impacts of shell recycling are primarily associated with energy-intensive calcination processes at high temperatures [28]. Therefore, the direct acid–carbonate neutralization route employed in this work, which avoids calcination and external heating, may reduce process complexity, energy consumption, and associated CO_2_ emissions. In addition, the utilization of shell waste as a calcium source may contribute to waste valorization and potentially reduce raw material costs.

## 3. Materials and Methods

### 3.1. Materials and Reagents

Cockle shells (*Anadara granosa*) were collected from local seafood processing waste in Thailand. The collected shells were thoroughly washed with deionized water to remove organic matter and dried at 105 °C for 24 h. The dried cockle shells were crushed and ground with a mechanical grinder, then sieved to obtain particles smaller than 150 µm. The obtained powder, mainly composed of calcium carbonate (CaCO_3_), was stored in a desiccator before use. Propionic acid (99.5% Sigma-Aldrich, Saint Louis, MO, USA) with different concentrations (80%, 90%, and 99%) was used as the reacting acid. All chemicals were of analytical grade and used as received without further purification. Deionized water was employed throughout the experiments.

### 3.2. Synthesis of Calcium Propionate

Calcium propionate was synthesized via a direct acid–base reaction between calcium carbonate derived from cockle shells and propionic acid of varying concentrations (80%, 90%, and 99%). In a typical experiment, shell-derived CaCO_3_ powder was used as a green calcium source, and the molar ratio of CaCO_3_ to propionic acid was fixed at 1:2. Based on this ratio, a calculated amount of CaCO_3_ (98% purity, typically 5.00 g, corresponding to 0.050 mol) was gradually added to the required amount of propionic acid (0.100 mol) under continuous magnetic stirring (IKA, Staufen, Germany) at ambient temperature. The molar ratio of CaCO_3_ to propionic acid was controlled according to the stoichiometric reaction Equation (4):

CaCO_3_ + 2 CH_3_CH_2_COOH → Ca(CH_3_CH_2_COO)_2_ + CO_2_ + H_2_O (4)

The reaction was allowed to proceed until complete cessation of CO_2_ evolution, indicating full consumption of CaCO_3_. The reaction time was recorded to evaluate the effect of acid concentration on synthesis efficiency. The resulting products were subsequently dried in a hot-air oven at 60 °C until a constant weight was achieved. The solid product obtained was gently ground and stored in airtight containers for further characterization.

### 3.3. Characterization

#### 3.3.1. Thermogravimetric Analysis (TGA)

The thermal stability and decomposition behavior of calcium propionate samples were investigated using thermogravimetric analysis (TG/DTA, Pyris Diamond, PerkinElmer, Waltham, MA, USA). The measurements were carried out in a nitrogen atmosphere from room temperature to 800 °C at a heating rate of 10 °C min^−1^. Weight-loss profiles were used to assess the moisture content, organic decomposition, and thermal characteristics of the products synthesized at different propionic acid concentrations.

#### 3.3.2. Fourier Transform Infrared Spectroscopy (FTIR)

Fourier transform infrared (FTIR) spectra of the prepared calcium propionate samples were recorded using an FTIR spectrometer (Spectrum GX, PerkinElmer, Waltham, MA, USA) in the range of 4000–400 cm^−1^. The samples were analyzed using the KBr pellet method. The characteristic absorption bands were used to confirm the formation of calcium propionate and to compare products obtained from different propionic acid concentrations.

#### 3.3.3. X-Ray Diffraction (XRD)

The crystalline structure of the synthesized products was examined by X-ray diffraction (MiniFlex, Rigaku Corporation, Tokyo, Japan) using Cu Kα radiation (λ = 1.5406 Å). Data were collected over a 2θ range of 5–60° at room temperature. The obtained diffraction patterns were compared with standard reference data to identify the crystalline phase of calcium propionate and to evaluate structural differences arising from varying acid concentrations.

#### 3.3.4. Scanning Electron Microscopy (SEM)

The surface morphology and microstructure of the synthesized calcium propionate were observed using scanning electron microscopy (SEM, VP1450, LEO, North Billerica, MA, USA). Prior to analysis, the samples were coated with a thin layer of gold to improve conductivity. SEM images were used to examine particle size, shape, and agglomeration behavior, and the results were compared with those reported for other calcium salts prepared from shell biowaste in our earlier works.

#### 3.3.5. X-Ray Fluorescence Spectroscopy (XRF)

Elemental composition of the synthesized calcium propionate was analyzed using X-ray fluorescence spectroscopy (Bruker AXS, Billerica, MA, USA). Samples were prepared as pressed powder pellets and placed directly into the spectrometer sample chamber. The potential used was 30 kV and a current of 40 µA, with an exposure of 180 s for each sample. The analysis was performed to quantify the calcium content and to detect possible impurities originating from the biowaste shells. This technique was applied using the same analytical strategy as in our previous studies of calcium-based compounds.

## 4. Conclusions

Calcium propionate was successfully synthesized from cockle shell-derived CaCO_3_ via a direct reaction with propionic acid at concentrations of 80%, 90%, and 99%. This work presents two key aspects of novelty. First, to the best of our knowledge, this is the first reported synthesis of calcium propionate using cockle shell biowaste as a biogenic calcium source. Second, unlike conventional approaches employing dilute acid concentrations of 10–15%, the use of highly concentrated propionic acid significantly shortened the reaction time, improved the overall product yield beyond previously reported values, and enabled the synthesis to proceed without external heating, offering a less complex production route than in previous studies. All prepared samples were confirmed to consist of calcium propionate monohydrate (Ca(CH_3_CH_2_COO)_2_·H_2_O), as evidenced by the FTIR, XRD, TGA, and SEM analyses. XRF confirmed more than 97%wt CaO across all samples with non-toxic impurities corresponding to compositional requirements for food additive calcium propionate (E282). Among the conditions investigated, 80% propionic acid showed the best results, achieving the highest yield (90.24%) and soluble percentage (98.23%). This study highlights a sustainable and practical route to produce the food additive calcium propionate (E282) from renewable biogenic resources, with potential for contributing to waste valorization, cost reduction, and the advancement of circular economic practices in food additive production.

## Figures and Tables

**Table 1:** Elemental compositions of calcium propionate derived from cockle shells using propionic acid with different concentrations (80%, 90%, and 99%), denoted as CPN-80, CPN-90, and CPN-99, respectively.

Compounds	Formula	Chemical Contents/wt%		
		CPN-80	CPN-90	CPN-99
Calcium oxide	CaO	97.26	97.21	97.26
Disodium oxide	Na_2_O	1.17	1.23	1.17
Magnesium oxide	MgO	0.41	0.41	0.41
Aluminum oxide	Al_2_O_3_	0.36	0.40	0.36
Silicon dioxide	SiO_2_	0.37	0.31	0.37
Phosphorous oxide	P_2_O_5_	0.21	0.22	0.21
Sulfur oxide	SO_3_	0.13	0.12	0.13
Dipotassium oxide	K_2_O	0.04	0.05	0.04
Ferric oxide	Fe_2_O_3_	0.03	0.03	0.03
Strontium oxide	SrO	0.02	0.02	0.02
**Total**		**100.00**	**100.00**	**100.00**

**Table 2:** Reaction time, production yield, and solubilities of calcium propionate derived from cockle shells using propionic acid with different concentrations (80%, 90%, and 99%), denoted as CPN-80, CPN-90, and CPN-99, respectively.

Samples	Propionic Acid Concentration /%*w*/*w*	Reaction Temperature /°C	Reaction Time/h	Yield/%	Soluble Percentage/%
CPN-80	80	33	12	90.24	98.23
CPN-90	90	37	9	83.09	96.54
CPN-99	99	42	5	78.55	91.81

## Data Availability

All data are fully available without restriction.
